# Longitudinal Characterization of Transcriptomic, Functional, and Morphological Features in Human iPSC-Derived Neurons and Their Application to Investigate Translational Progranulin Disease Biology

**DOI:** 10.3389/fnagi.2020.576678

**Published:** 2020-11-12

**Authors:** Gaëlle Robin, J. Corey Evans, David N. Hauser, Paul Wren, Andreas Zembrzycki

**Affiliations:** ^1^SBP-GSK Center for Translational Neuroscience, Sanford Burnham Prebys Medical Discovery Institute, La Jolla, CA, United States; ^2^GSK, Neuroscience Discovery, Collegeville, PA, United States

**Keywords:** progranulin (PGRN), frontotemporal lobe dementia (FTD), neurodegeneration, translational neuroscience, MEA, RNA-Seq, live-cell imaging

## Abstract

The disease biology of frontotemporal lobe dementia (FTD) is complex and not fully understood, with limited translational value appreciated from animal models to date. Human cellular systems that can recapitulate phenotypic features of disease offer promise as translational tools to not only increase our understanding of disease processes but also increase the probability of success of translating novel treatment options to patients. However not all researchers may necessarily have access to well-characterized induced pluripotent stem cell (iPSC)-derived human neurons. As an example, we therefore comprehensively profiled phenotypic features over time in one commercially-available IPSC-derived human neuron cell line. This included systems-level assessments of neurite outgrowth dynamics, neuronal network function, and genome-wide gene expression. By investigating progranulin biology as an example we then demonstrated the utility of these cells as a tool to investigate human disease biology. For example, by using the siRNA-mediated knockdown of the progranulin (*GRN*) gene, we demonstrated the establishment of an isogenic human cellular model to facilitate translational FTD research. We reproduced findings from rodent neurons by demonstrating that recombinant progranulin (rPGRN) mediated neuroprotection. Contrary to previous rodent data, in our human cellular models, growth factor treatment showed no consistent sensitivity to modulate neurite outgrowth dynamics. Our study further provides the first evidence that rRPGRN modulated neuronal firing and synchrony in human neurons. Taken together, our datasets are a valuable systems-level resource demonstrating the utility of the tested commercially-available human iPSC neurons for investigating basic human neurobiology, translational neuroscience, and drug discovery applications in neurodegenerative and other CNS diseases.

## Introduction

While most basic biological studies utilize animal-based models to investigate disease mechanisms and biology, suitable models that allow translation of these findings to human biology are often missing. This is especially true in the neuroscience field, which lacks broadly available well-characterized human neuronal models. Variability in pharmacological responsiveness between animal and human cells is particularly rate-limiting for quantitative drug discovery applications and the identification of potential therapeutic molecules. Therefore, novel human cell-based model systems could offer key advantages for translational biology. Induced pluripotent stem cells (iPSCs) can be differentiated into a host of cell types, including neurons (Takahashi et al., 2007) and consequently, have been implemented to model common and rare neurological diseases (Park et al., 2008; Ross and Akimov, 2014), including Alzheimer’s disease (AD; Israel et al., 2012) and frontotemporal lobe dementia (FTD; Almeida et al., 2012; Raitano et al., 2015). Such iPSC-derived neuronal models from normal donors and patients are useful but have certain disadvantages. The creation of these cells is time-consuming and requires extensive characterization (Xu and Zhong, 2013); moreover, results have been shown to vary substantially between labs (Volpato et al., 2018).

Commercially-available iPSC-derived neurons are already differentiated and subjected to quality control, thus making this technology available to the broader research community that would otherwise need expertise in iPSC reprogramming and differentiation procedures. Well-characterized commercially-available cells would be a useful alternative to enhance the availability and reproducibility of iPSC models. iPSC-derived neurons representing distinct brain regions are commercially-available, including the iCell^®^ line from FUJIFILM Cellular Dynamics, Inc. (FCDI). iCell^®^ GlutaNeurons (GNs), for example, express predominantly glutamatergic markers and according to the manufacturer have been previously used to study electrophysiology, cell survival, and neurite outgrowth and the neuronal identity of GNs has been confirmed by flow cytometry and by assessment of gene expression in individual cells, while neuronal function has been assayed by multi-electrode array and calcium influx analyses (Fujifilm, 2018). Therefore, our objective was to perform an independent and longitudinal assessment of key phenotypic features in human iPSC-derived GNs Using live-cell imaging, RNA-Sequencing (RNA-Seq), and multi-well multi-electrode array (mwMEA) technologies, we present comprehensive data characterizing: (1) neuronal morphology and outgrowth; (2) neuronal network activity and synchrony; and (3) genome-wide gene expression profiles in GNs over a culture period of 3 weeks.

Next, we used the cells to study progranulin (PGRN) as proof-of-principle that these human neuronal cells are applicable to better understand translational disease biology in the context of neurodegenerative disease. FTD is a cluster of neurological disorders associated with behavioral and speech abnormalities and the most common pre-senile dementia (Bang et al., 2015). Gene linkage studies identified that heterozygous mutations in the *GRN* gene lead to haploinsufficiency of the encoded PGRN protein and cause FTD characterized by prominent deterioration of frontal and temporal cortical lobes (Baker et al., 2006; Cruts et al., 2006). We developed a siRNA-mediated knockdown protocol to study how the loss of endogenous *GRN* expression in human iPSC neurons relates to *GRN* haploinsufficiency in human FTD patients and studied how the treatment of the used human iPSC neuronal cultures with recombinant PGRN impacts neuronal outgrowth dynamics, resilience towards neurotoxic stress and neuronal function. This work aimed to explore the feasibility of using commercially-available human IPSC glutamatergic neurons as a tool to investigate neuronal structure and function concerning disease with direct application to understanding the influence of progranulin, a common causal genetic risk factor for FTD (Baker et al., 2006; Cruts et al., 2006) on relevant phenotypic endpoints.

## Materials and Methods

### Culture of iPSC-Derived Neurons

iCell^®^ GlutaNeurons (GNs; FUJIFILM Cellular Dynamics Inc., FCDI) are human cells differentiated from a master bank of stably induced pluripotent stem (iPS) cells from normal donors (stemcell.com). The iPS cell lines were generated from human peripheral blood through ectopic expression of reprogramming factors (i.e., Oct4, Sox2, Nanog, Lin28, Klf4, L-Myc, and SV40LT) by episomal transfection. The iPS cell clones were then engineered using nuclease-mediated methodologies to exhibit neomycin resistance under the control of a neuronal-specific promotor. GNs were cultured according to the manufacturer’s protocol. Clear, flat-bottom 96-well plates (Corning) were coated with 0.01% poly-L-ornithine (PLO, Sigma–Aldrich) for a minimum of 1 h at room temperature. After three washes with PBS (Thermo Fisher Scientific, Waltham, MA, USA), the wells were coated with 0.028 mg/ml Growth Factor Reduced Matrigel (Corning) for at least 1 h in a 37°C incubator. Cells were thawed for 2 min in a 37°C water bath, then slowly equilibrated by the dropwise addition of room-temperature complete medium [BrainPhys Neuronal Medium (STEMCELL Technologies) containing iCell^®^ supplements (CDI), N-2 Supplement (Thermo Fisher Scientific, Waltham, MA, USA), laminin (1 μg/ml, Sigma–Aldrich), and penicillin-streptomycin (Thermo Fisher Scientific, Waltham, MA, USA)]. The cells were then plated and allowed to recover overnight at 37°C.

For culturing GNs for multielectrode array (MEA) experiments, 48- and 96-well MEA plates (Axion Biosystem, Atlanta, GA, USA) were coated with 80 μl of 0.07% polyethyleneimine (Sigma–Aldrich) diluted in borate buffer for 1 h at room temperature. The plates were then washed four times with sterile water and air-dried overnight in a sterile biological safety cabinet. GNs were thawed as described above and were resuspended in dotting medium (Complete BrainPhys Medium with 11 μg/ml laminin) to reach a concentration of 10 million cells/ml and 7 million cells/ml for 48- (M768-KAP-48) and 96-well plates (M768-KAP-96), respectively. A 10 μl droplet of the cell suspension was then manually dispensed directly over the recording electrode area of each well. Plates were kept for 1 h in the incubator at 37°C before gently adding 300 μl of fresh complete BrainPhys Medium in each well. A 50% medium change was performed 1-day post-plating and then every other day.

### IncuCyte™ ZOOM Analysis

We utilized the IncuCyte™ ZOOM Live-Cell Analysis System (Essen BioScience) to perform live imaging assays for neurite outgrowth and cellular toxicity. This system consists of an incubator housing a microscope for the acquisition of phase as well as red/green fluorescent images over a user-defined time range. By leveraging the systems software modules and algorithms, we performed rapid, long-term, and unbiased high-throughput analyses of multiple morphological features in real-time. Using the NeuroTrack™ module (Essen BioScience), a set of criteria (called a “processing definition”) defining cell bodies and neurites was optimized for GNs to quantify neuronal morphology metrics, including neurite length, the number of neurite branch points, and the number of cell body clusters.

For neurite outgrowth analysis, the following parameters were used for the NeuroTrack™ software: Cell-Body Cluster Parameters = Segmentation Mode (Brightness), Segmentation Adjustment (0.2); Cleanup = Hole Fill (0 μm^2^), Adjust Size (0 pixels), Min Cell Width (9 μm); Cell-Body Cluster Filters = Area (min = 290 μm^2^); Neurite Parameters = Filtering (None), Neurite Sensitivity (0.55), Neurite Width (1 μm). For monitoring cellular morphology during siRNA treatment, the following parameters were used for the NeuroTrack™ software: Cell-Body Cluster Parameters = Segmentation Mode (Brightness), Segmentation Adjustment (0.9); Cleanup = Hole Fill (0 μm^2^), Adjust Size (0 pixels), Min Cell Width (7 μm); Cell-Body Cluster Filters = None; Neurite Parameters = Filtering (None), Neurite Sensitivity (0.5), Neurite Width (1 μm).

For Annexin V analysis, the following parameters were used for the Basic Analyzer™ software: Phase parameters = Segmentation Adjustment (0.4), Cleanup = Hole Fill (0 μm^2^), Adjust Size (0 pixels), Min Area (40 μm^2^); Green Channel (Annexin V) parameters = Top hat Radius (60 μM), Top hat Threshold (2 GCU), Edge split on, Edge split sensitivity (0), Cleanup = Hole Fill (0 μm^2^), Adjust Size (0 pixels).

### Neurite Outgrowth Assay

**Figure 1:**
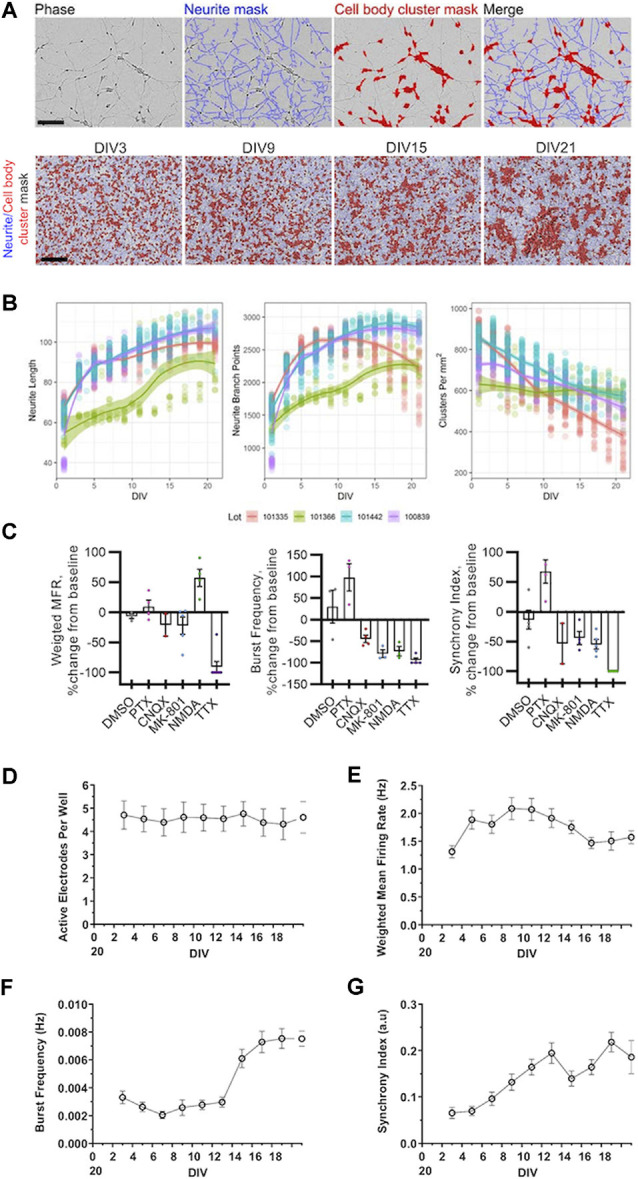
Morphological and electrophysiological characterization of cultured human induced pluripotent stem cell (iPSC) neurons. **(A)** Representative phase-contrast images of GlutaNeuron (GN) neurite and cell body cluster masking by NeuroTrack™ software at indicated day *in vitro* (DIV), with neurite masks shown in blue and cell-body cluster masks shown in red. Scale bar, 600 μm. **(B)** Quantification of neurite length, neurite branch points, and cell-body clusters in cultured GNs from four different lots. Graphs represent data from 12 independent experiments (*n* = 198 wells total) with 1,452 data points per variable (516 for Lot 101335, 123 for Lot 101366, 453 for Lot 101442, and 360 for Lot 100839). Solid lines show local regression (LOESS) models of the data along with 95% confidence intervals. **(C)** Weighted mean firing rate (MFR), burst frequency, and the synchrony index were measured using multi-well multielectrode array (mwMEA) recordings at baseline and after the addition of vehicle (DMSO) and established neuronal firing modulators 50 μM picrotoxin (PTX), 10 μM CNQX, 1 μM dizocilpine (MK-801), 20 μM NMDA, 1 μM tetrodotoxin (TTX). **(D–G)** Quantification of neuronal activity in GNs from DIV3 to DIV21. **(D)** Active electrodes per well over 21 days in culture. **(E)** Weighted MFR over 21 days in culture, normalized to weighted MFR measured at 3 DIV. **(F)** Burst frequency over 21 days in culture, normalized to burst frequency recorded at 3 DIV. **(G)** Synchrony index over 21 days in culture, normalized to synchrony index recorded at 3 DIV. mwMEA graphs represent data from seven independent experiments (8–24 replicates per condition in each experiment), and the results are displayed as the mean ± standard deviation (SD).

One day after plating (7,000 cells/well), GNs were treated with either PBS (vehicle), 7.41 nM BDNF (PeproTech), 7.41 nM NGF (R&D Systems), concentrations that in previous studies were sensitive to increase neurite outgrowth (Van Damme et al., 2008) or (Thermo Fisher Scientific, Waltham, MA, USA). For all samples, the amount of the vehicle was kept the same. After 50% of the complete medium was removed from the cells, the aforementioned treatments (prepared as 2× concentrations) were diluted 1:1 in the remaining medium. Every other day, half of the medium in each well was exchanged with fresh medium containing the appropriate treatments. The cells were imaged using the IncuCyte™ ZOOM Live-Cell Analysis System (beginning approximately 1 h after the initial treatment); neurite morphology was assessed on the 10th day of treatment. Representative images show that an optimized NeuroTrack™ processing definition accurately masked neurites for quantification and analysis (Figure 4E). Both neurite length and the number of branch points increased over the 10-day assay, as represented by GNs treated with vehicle (PBS; Figure 4F); this is consistent with the increased neurite growth over time seen in GNs plated at a higher density in previous experiments (Figures 1B,C). To determine the fold-change in the neurite metrics, all measurements at day 10 of treatment were normalized to those of the first imaging time point (i.e., 1 h after the first treatment).

**Figure 2:**
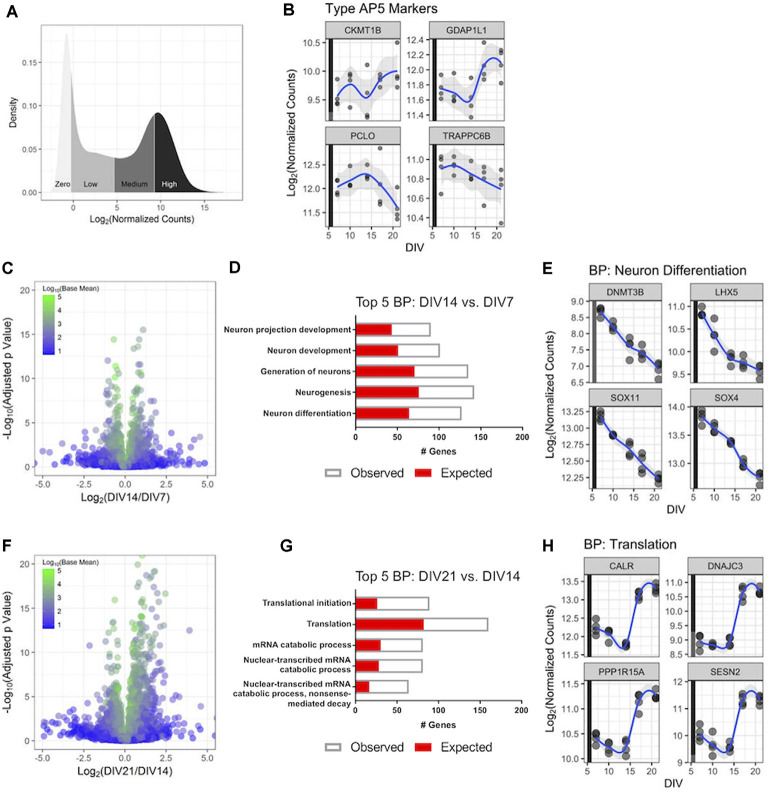
RNA-Sequencing(RNA-Seq) profiling of human iPSC neurons during 3 weeks in culture. Untreated GN cultures were collected over 3 weeks in culture, and global gene expression was determined with RNA-Seq. **(A)** Distribution of the RNA-Seq count data for the 27,882 genes measured in the experiment. The *y*-axis displays the kernel density estimate, and the *x*-axis displays the normalized count data with each quartile shaded. **(B)** Expression of four genes associated with highly functional (Type AP5) iPSC-derived neurons over time. Each point represents an independent replicate (*n* = 4 independent replicates per time point), and the blue line shows a LOESS model of the data. The colored stripe on the *y*-axis indicates the quartile of count values shown in panel **(A)**. **(C)** Volcano plot displaying RNA-Seq data comparing gene expression at DIV14 relative to DIV7. The plot displays 16,591 genes colored according to the mean of their normalized count values across all samples (*n* = 4 independent replicates per time point). **(D)** The top five biological processes (BPs)—identified by gene ontology (GO) analysis—that were significantly enriched with differentially expressed genes at DIV14 vs. DIV7. The number of genes observed as differentially expressed and the number expected by chance are superimposed. **(E)** Expression values over time for the four most statistically significant genes in the neuron differentiation BP at DIV14 compared to DIV7. Data are displayed as in panel **(B)**. **(F)** Volcano plot of RNA-Seq data comparing gene expression at DIV21 and DIV14 displayed as in panel **(C)**. **(G)** The top five BPs that were significantly enriched with differentially expressed genes at DIV21 compared to DIV14. **(H)** Expression values over time for the four most statistically significant genes in the translation BP at DIV21 compared to DIV14. Data are displayed as in panel **(B)**.

**Figure 3:**
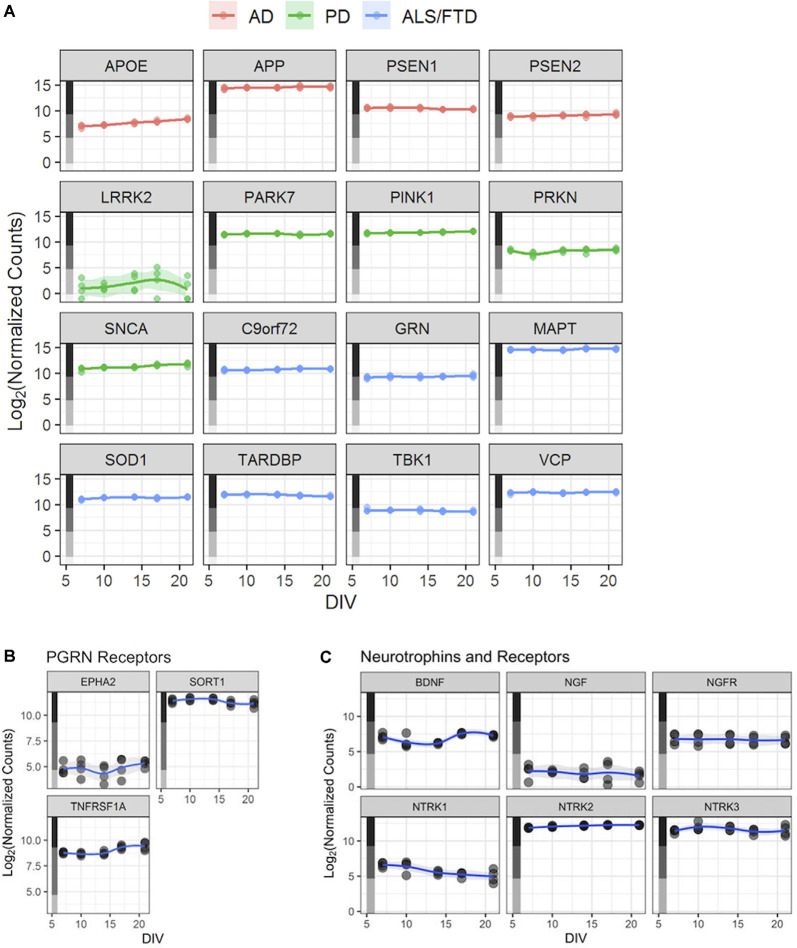
Human iPSC neurons as a potential tool to study neurodegenerative and other CNS diseases. **(A)** Expression profiles—based on the RNA-Seq dataset described in Figure 2—for genes linked to selected neurodegenerative diseases. Each point represents an independent replicate (*n* = 4 independent replicates per time point), and the line for each graph shows a LOESS model of the data. The colored stripe on the *y*-axis indicates the quartile of count values shown in Figure 2A. Alzheimer’s Disease (AD), Parkinson’s Disease (PD), amyotrophic lateral sclerosis (ALS), frontotemporal lobe dementia (FTD). **(B)** Expression of three PGRN receptor genes—*EPHA2*, *SORT1*, and *TNFRSF1A—*in untreated GNs. **(C)** Expression of *BDNF*, *NGF*, and their receptors—*NGFR* (NGF receptor gene) and *NTRK1–3* (BDNF receptor genes) in untreated GNs. Data in panels **(B,C)** are displayed as in Figure 2B.

**Figure 4:**
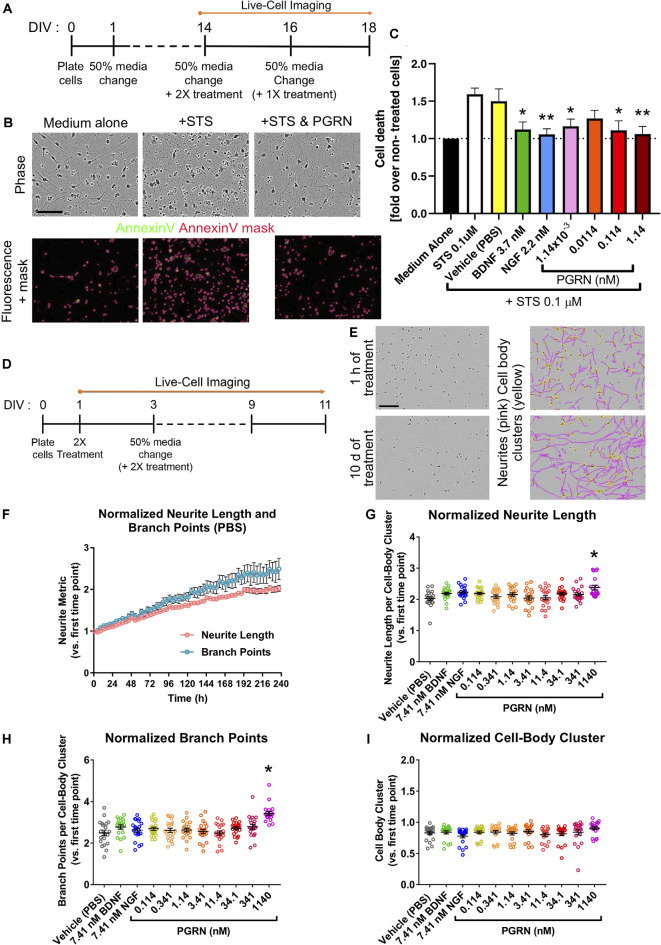
Recombinant progranulin (PGRN) mediates neuroprotection in human iPSC neurons. **(A)** Schematic of the neurotoxicity assay timeline. Dashes represent 50% of media changes every other day. **(B)** Representative images of DIV16 GNs 48 h after either medium change alone (Medium alone), staurosporine (STS) treatment alone (+STS), or STS and 0.114 nM PGRN co-treatment (+STS and PGRN). The top panel shows phase-contrast images of the cells; the bottom panel displays fluorescence images showing the annexin V staining in the green channel with the superimposed annexin V mask (in pink). This user-defined mask was generated using the Basic Analyzer software module in the IncuCyte™ system and was used to quantify cell death based on annexin V fluorescence signal. Scale bar, 400 μm. **(C)** Measured by annexin V staining-covered area, graph shows the fold change of cell death and toxicity concerning untreated wells. Cells were untreated (medium alone) or co-treated with STS and recombinant PGRN, BDNF, or NGF. The graph represents data from 4 or 10 independent experiments (three replicates per condition in each experiment). Results are displayed as mean ± SD. For all graphs, *p < 0.05; **p < 0.01 (one-way ANOVA with Dunnett’s *post hoc* test, all conditions compared to STS treated wells). **(D)** Schematic of the neurite outgrowth assay. Dashes represent 50% media changes (containing treatments in double concentration: 2×) every other day. **(E)** Representative phase-contrast images and the masking of neurites utilizing the NeuroTrack™ software in the IncuCyte™ system. Images and masks were used to quantify neurite length and neurite branch points. Example images show PBS vehicle-treated GNs; images show masked neurites (pink) and cell body clusters (yellow) after 1 h and after 10 days of treatment. Scale bar, 600 μm. **(F)** Quantification of neurite length and neurite branch point measurements for vehicle-treated GNs. Each data point represents the mean ± SEM for four to five independent experiments (four replicates per experiment). **(G–I)** Quantification of neurite metrics after 10 days of treatment with either vehicle (PBS), 7.41 nM BDNF, 7.41 nM NGF, or a range of PGRN concentrations. All graphs represent data from five independent experiments (four replicates per condition in each experiment). Results are displayed as mean ± SEM. For all graphs, *p < 0.0001 (one-way ANOVA with Dunnett’s *post hoc* test, all conditions compared to vehicle). **(G)** Neurite length per cell-body cluster (normalized to the measurement at the first imaging time point). **(H)** Neurite branch point number per cell-body cluster (normalized to the measurement at the first imaging time point). **(I)** Cell-body cluster number (normalized to the measurement at the first imaging time point).

### Neurotoxicity Assay

GNs plated at 25,000 cells/well were treated at DIV14 with IncuCyte^®^ Annexin V Green (4642, Essen BioScience Inc.,) at a final dilution of 1:200. Annexin V, a Ca^2+^-dependent phospholipid-binding protein, has a high affinity for membrane phosphatidylserine, which translocates from the inner side of the plasma membrane to the cell surface during the early stages of apoptosis (Andree et al., 1990; Koopman et al., 1994). Neuronal toxicity was induced by treating the cells with staurosporine, a nonselective protein kinase inhibitor (STS, Enzo Life Sciences) at 0.1 μM. Vehicle (PBS, Thermo Fisher Scientific, Waltham, MA, USA), 2.2 nM recombinant NGF (Human β-NGF, R&D Systems), 3.7 nM recombinant BDNF (R&D Systems) or a concentration range of PGRN Recombinant Human Progranulin/PGRN (R&D Systems, Thermo Fisher Scientific, Waltham, MA, USA) was co-added to the STS treatment as indicated. For all samples, the amount of the vehicle was kept the same. After 50% of the complete medium was removed from the cells, the treatments mentioned above (prepared as 2× concentrations) were diluted 1:1 in the remaining medium. Every other day, half of the medium in each well was exchanged with fresh medium containing the appropriate treatments. The cells were imaged using the IncuCyte™ ZOOM Live-Cell Analysis System (Essen BioScience; beginning approximately 1 h after the initial treatment).

### siRNA-Mediated Gene Knockdown

The Accell Human *GRN* siRNA SMARTpool (E-009285-00-0050) and Accell Non-targeting Pool (D-001910-10-50) were purchased from Dharmacon. The target sequences of the *GRN* SMARTpool are GUGCUGUGUUAUGGUCGAU, GAGAUGUCCCCUGUGAUAA, UUGUCCAGCUCGGUCAUGU, and GUGCGUUUCAAUAAAGUUU. The target sequences of the Non-targeting Pool are UGGUUUACAUGUCGACUAA, UGGUUUACAUGUUUUCUGA, UGGUUUACAUGUUUUCCUA, and UGGUUUACAUGUUGUGUGA. The siRNA was reconstituted to 100 μM with 1× Dharmacon siRNA Buffer (B-002000-UB-100) and added directly to the GN media immediately before use. All experiments using siRNA were done with 70,000 GNs per well in 96-well plates. The first 50% media change on the day *in vitro* (DIV) three was done with 2× concentrated siRNA to achieve the desired concentration of siRNA in each well. All subsequent 50% of media changes were done with freshly added 1× siRNA to maintain the desired siRNA concentration during the culture.

### RNA-Sequencing (RNA-Seq)

Plates containing GNs were washed with PBS and then frozen at −80°C until they were thawed for RNA purification using the RNEasy UCP Micro Kit (Qiagen #73934). Four independent experiments were performed (two using lot 100839 and two using lot 101442), and there were five to six technical replicate wells per condition per experiment. The lysates from technical replicate wells were combined before RNA purification to yield sufficient quantities of RNA for RNA-Seq. PolyA RNA was isolated using the NEBNext^®^ Poly(A) mRNA Magnetic Isolation Module, and barcoded libraries were made using the NEBNext^®^ Ultra II™ Directional RNA Library Prep Kit for Illumina^®^ (NEB, Ipswich, MA, USA). Libraries were pooled and single-end sequenced (1 × 75) on the Illumina NextSeq 500 using the High output V2 kit (Illumina Inc., San Diego, CA, USA). Transcript level quantification was generated using *Salmon* (v0.8.2; Patro et al., 2017), and an index was built using cDNA sequences from the human genome assembly GRCh38 (ftp://ftp.ensembl.org/pub/release-92/fasta/homo_sapiens/cdna/Homosapiens.GRCh38.cdna.all.fa.gz). Gene abundance estimates were created with *Tximport* (v1.8.0; Soneson et al., 2015) using a tx2gene file containing Ensembl transcript IDs and their associated HGNC symbols. Samples were assigned to one of fifteen possible groups based on treatment type and day *in vitro* (DIV; Untreated_DIV7, *GRN*_siRNA_DIV7, Control_siRNA_DIV7, et cetera), and a DESeqDataSet was constructed using a design formula of ~group in *DESeq2* (Love et al., 2014). Genes with count means of less than five were removed before principal component analysis (PCA) and pairwise comparisons. Differential expression was determined using the *DESeq2* default of a Benjamini–Hochberg adjusted *p-value* cutoff of 0.10. Gene ontology (GO) analysis was performed using the Overrepresentation Enrichment Analysis method of WebGestalt (Wang et al., 2017) with “geneontology” selected as the functional database. Note that the RT-PCR presented in Figure 7B is from the same samples we used for RNA-Seq. The RT-PCR data shows reduced GRN expression in treated samples. This validation our RNA-Seq data, which similarly demonstrated reduced GRN expression through an orthogonal method. RNA-Seq data are publicly accessible at the Gene Expression Omnibus under Accession GSE157573.

**Figure 5:**
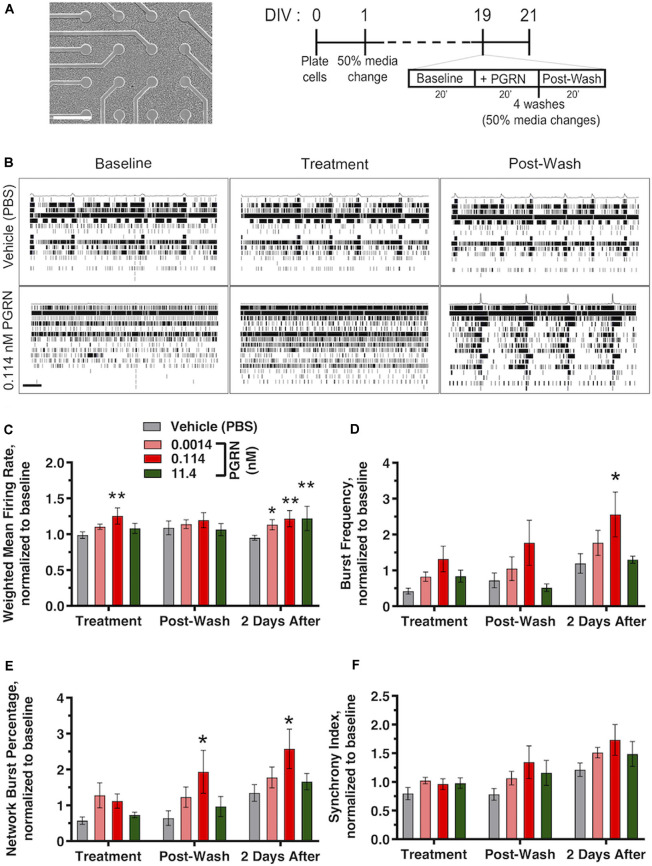
Recombinant PGRN enhances firing and synchronization in cultured human iPSC neurons. **(A)** Representative images of GNs at 19 DIV growing on top of the electrodes of a 48-well mwMEA plate and schematic of the experiment and mwMEA recording. Dashes represent 50% of media changes every other day. Recordings were made before, during, and after PGRN treatment on DIV19; then, an additional recording was performed 2 days later. **(B)** Representative raster plots for neuronal firing before, during, and after exposure to the vehicle (PBS) or 0.114 nM PGRN in DIV19 cells. Each row of the raster plot represents an electrode within a well (16 total), and each black line within the row represents a neuronal spike. Averages of spikes are shown at the top of each raster plot. Scale bar, 20 s. **(C–F)** Quantification of neuronal activity relative to baseline activity in cells during treatment, directly after washes, and 2 days after treatment/washed. All graphs represent data from six independent experiments (two to six replicates per condition in each experiment). Results are displayed as mean ± standard error of the mean (SEM). For all graphs, *p < 0.05; **p < 0.01 (one-way ANOVA with Dunnett’s *post hoc* test, all conditions compared to vehicle). **(C)** Weighted mean firing rate (MFR), normalized to baseline weighted MFR recorded before treatments. **(D)** Burst frequency, normalized to baseline burst frequency recorded before treatments. **(E)** Network burst percentage, normalized to baseline network burst percentage recorded before treatments. **(F)** Synchrony index, normalized to baseline synchrony index recorded before treatments.

**Figure 6:**
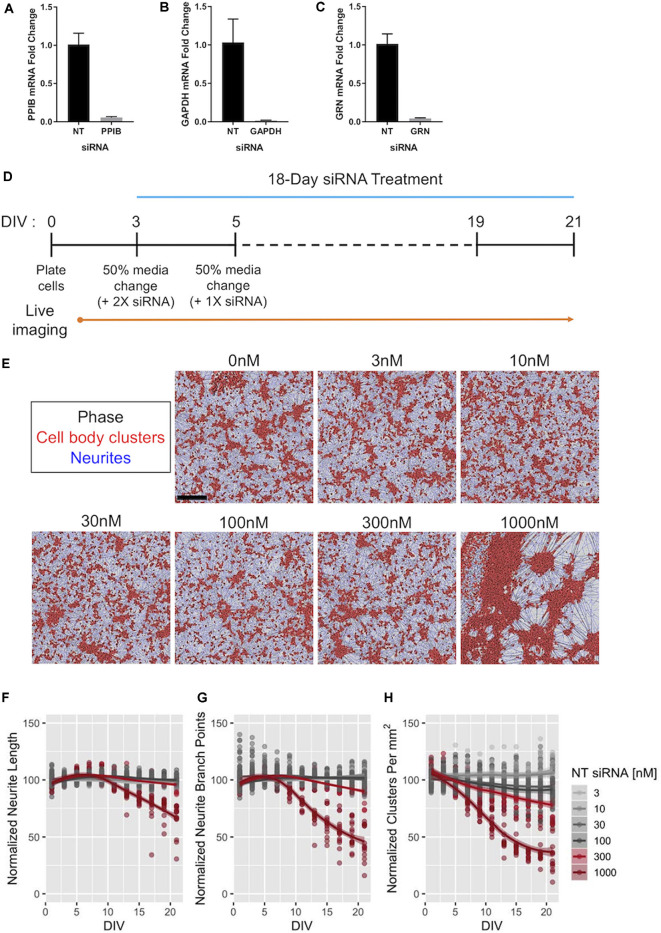
Efficacy of Accell™ siRNA-mediated gene knockdown in human iPSC neurons. **(A–C)** Expression of *PPIB* **(A)**, *GAPDH* **(B)**, and *GRN* **(C)** mRNA in GNs after 72-h exposure to 1 μM of siRNAs targeting the indicated gene. The expression levels were compared to those in GNs treated with 1 μM of pooled non-targeting (NT) siRNAs. Expression was detected using qPCR with 18S rRNA as an internal control (*n* = 3 independent replicates per group). **(D)** Schematic of the protocol for long-term treatment of self-delivering Accell™ siRNAs to GNs. Dashes represent 50% media changes (with 1× siRNA treatments) every other day. **(E)** Representative images taken at DIV21 of GNs repeatedly exposed to the indicated concentrations of NT siRNA per the protocol in panel **(D)** neurite masking by NeuroTrack™ software is shown for neurites (blue) and cell-body clusters (red). Scale bar, 300 μm. **(F–H)** NeuroTrack™ quantification of neurite length **(F)**, neurite branch points **(G)**, and cell-body clusters **(H)** in cells treated with the indicated concentrations of NT siRNA until DIV21. The data were collected for nine independent experiments (*n* = 183 untreated wells, *n* = 120 wells treated with 30 nM, and *n* = 15 wells for remaining concentrations). All measurements were normalized to the mean of the untreated cells per recording for each experiment. Solid lines show a LOESS fit of the data, and the shaded area represents the 95% confidence intervals of the model (when large enough to display). The concentrations displayed in red were significantly different than untreated cells at DIV21 [*p*_adj_ < 0.05, Tukey’s Honest Significant Difference (HSD) test].

**Figure 7:**
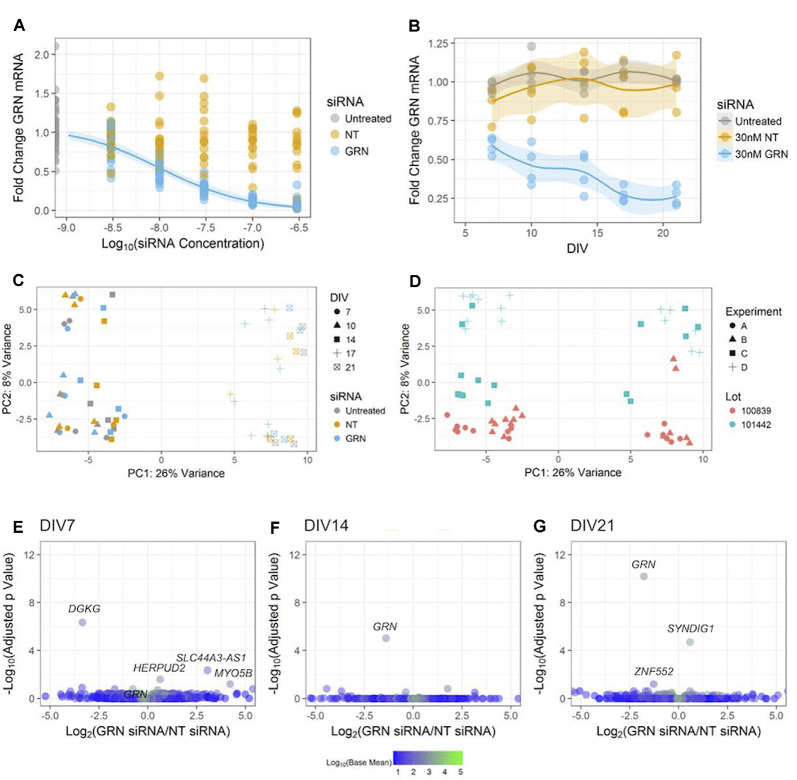
Reducing *GRN* expression in human iPSC neurons to model FTD. **(A)** *GRN* mRNA levels were determined using qPCR (with *ACTB* as a reference gene) following 18 days of treatment with the indicated siRNA concentrations. A three-parameter log-logistic model, with a bottom set to zero, was fit to the *GRN* siRNA data and is displayed as a blue line along with 95% confidence intervals. No model converged for the NT siRNA data. Untreated and *GRN* siRNA data come from seven independent experiments (*n* = 36 untreated wells and 105 *GRN* siRNA wells), while the NT siRNA data come from five independent experiments (*n* = 75 wells). Gene expression data are not displayed for the highest dose of siRNA used in Figures 5E–H due to toxicity. **(B)** *GRN* mRNA expression levels measured using qPCR in GNs treated with 30 nM siRNA and collected at DIV7, 10, 14, 17, and 21 (*n* = 4 independent replicates per time point for each treatment). The solid line shows a LOESS model of the data, with the 95% confidence intervals displayed in the shaded areas. The mean of *ACTB*, *PPIB*, and *GAPDH* expression was used for the reference. **(C,D)** Principle component analysis of RNA-Seq data from the cells treated with siRNA and collected at DIV7, 10, 14, 17, and 21 (*n* = 60 samples). The data are displayed according to shape (indicating the DIV) and color (indicating the siRNA) in **(C)**, while **(D)** shows the data with the shape indicating the experiment and the color indicating the cell lot number. **(E–G)** Volcano plots of RNA-Seq data comparing 30 nM of either NT or *GRN* siRNA at DIV7, 14, and 21. Each plot displays 16,591 genes colored according to the mean of their normalized count values across all samples (*n* = 4 independent replicates per siRNA). The labeled points were statistically significant (*p*_adj_ < 0.1), except for the *GRN* point labeled in the DIV7 plot.

### Quantitative Reverse Transcription PCR

Gene expression in siRNA dose-response experiments was determined using the TaqMan™ Gene Expression Cells-to-CT™ Kit (Invitrogen #AM1728). For the RNA-Seq samples, cDNA was synthesized from 10 ng of total RNA using Superscript III (Invitrogen #18080), and gene expression were analyzed using the standard TaqMan protocol. The TaqMan assays used were: *GRN* (Hs00963707_g1), *PPIB* (Hs00168719_m1), *ACTB* (Hs99999903_m1), and *GAPDH* (Hs99999905_m1). The fold change of *GRN* relative to reference genes (*ACTB* for dose-response experiments or the mean of *ACTB*, *PPIB*, and *GAPDH* for the RNA-Seq samples) was determined using the comparative Ct method. The R package *drc* was used for dose-response modeling (Ritz et al., 2015).

### Multi-well Multielectrode Array (mwMEA)

The neuronal electrical activity of cultured GNs was acquired using the Maestro MEA system (Axion Biosystem) according to the manufacturer’s guidelines. Briefly, 48- or 96-well plates were inserted in the Maestro machine (set up at 37°C and 5% CO_2_ without perfusion) and were recorded using AxIS software (Axion Integrated Studio, Axion Biosystem, version 2.4.2.13). mwMEA plates were composed of 48 wells or 96 wells, with each well containing an array of 16 or 8, respectively, individual embedded nano-textured gold microelectrodes (~40–50 μm diameter; 350 μm center-to-center spacing) with four integrated ground electrodes (Axion Biosystems). Treatment with the control compounds was performed at DIV18–23 with 20 min baseline recording followed by 20 min recording after the addition of 55 μl of a 7× concentration of either 50 μM picrotoxin (PTX, Tocris), 10 μM 6-cyano-7-nitroquinoxaline-2, 3-dione (CNQX, Sigma–Aldrich), 1 μM dizocilpine (MK-801, Sigma–Aldrich), 30 μM *N*-methyl-D-aspartic acid (NMDA, Sigma–Aldrich) or 1 μM tetrodotoxin (TTX, Tocris). For the acute addition of PGRN experiments, GNs were recorded from DIV18 to DIV24 depending on the network maturation, which can be quantified by a synchrony index of ~0.2 (see “Results” section below). To start, a 20 min baseline recording was performed on the 48-well mwMEA plate followed by the addition of 55 μl of a 7× concentration of PGRN (ranging from 0.0014 to 11.4 nM) or PBS. Another 20 min recording was made in the presence of the compound. Then, the compounds were washed out with four 50% media changes. This was followed by a 20 min post-wash recording. Finally, plates were recorded 2 days post-treatment.

For the *GRN* haploinsufficiency experiments, *GRN* or NT siRNA (30 nM) was added 3 days post-plating by replacing 50% of the medium with 2× siRNA, and a first mwMEA recording was performed the same day for 20 min. From there, neuronal activity of the treated cells was measured for 20 min every other day until DIV21.

The channels of the mwMEA were sampled simultaneously with a gain of 1,200× and a sampling rate of 12.5 kHz/channel. For all recordings, a Butterworth band-pass filter (200 Hz – 3 kHz) was applied along with an adaptive threshold spike detector set at 6× standard deviation. Data from the recordings were saved to two different file types simultaneously: a raw data file (*.raw file) that included all data, and an AxIS spike file (*.spk file) that included the spikes per electrode with a 1 s bin time. The Axis spike data file was then analyzed using Neural Metric tool software (Axion Biosystem, version 2.2.4) with an active electrode criterion of 5 spikes per min. For a single electrode burst, the Poisson Surprise algorithm was selected with a setting of 10. For the network burst, the Envelope statistic was chosen with a threshold factor of 3, a minimum inter-burst interval of 2,000 ms, a minimum electrode percentage of 50%, and a burst inclusion of 75%.

## Results

### Quantifying the Morphology and Neurite Dynamics of Human iPSC Neurons

First, we characterized key morphological processes in cultured GNs neurons starting at the time of plating. We tracked neurite dynamics in GNs from four separate cell lots over 3 weeks in culture using live-cell imaging. Representative images in Figure 1A illustrate the precision of the automatic capture of neurite metrics by our optimized algorithms and the typical morphological progression of the cultures over time from DIV3 to DIV21. As expected, we were able to track and quantify the outgrowth and branching of neurites over time. Additionally, we were able to identify small, discrete groups of cell bodies in younger cultures that over time tended to form larger continuous cell cluster organizations. In general, across multiple lots, we found that neurite length and branching rapidly increased during the first few days in culture and then began to plateau after around DIV7 (Figure 1B). The number of defined groups of cells (named cell-body clusters), on the other hand, tended to decrease over time (Figure 1B), indicating that the cells and their soma were surprisingly mobile and tended to organize into larger groups as the cultures aged. Notably, we found that, although all lots of GNs showed robust outgrowth of neurites, there was considerable within-lot variation in the overall performance of the GNs in our morphological assays. For example, one lot consistently showed inferior performance across metrics as compared to the three others (Figure 1). Taken together, our unbiased live-cell imaging approach allowed us to quantify morphological parameters in commercially-available human iPSC-derived GNs and demonstrated that these cells are a useful tool to study neurite dynamics and the quantitative pharmacology of molecules that potentially modulate this process.

### Quantifying Neural Network Activity in Human iPSC Neurons

Outgrowth and branching of cultured GNs suggest that individual neurons connect into networks. We thus expected that—once neurons extended neurites and connect with each other—spontaneous electrical activity would emerge in the cultures. To capture and quantify dynamics in extracellular membrane potentials in plated excitable GNs (Obien et al., 2015) longitudinally, we plated them on mwMEA.

First, we validated qualitatively that GNs are electrophysiologically active and sensitive to established chemical modulators using our standard plating and cell culture conditions. Our results demonstrate that in GN cultures treatment with the sodium channel blocker TTX, the glutamate receptor antagonist dizocilpine (MK-801), or the AMPA/kainate receptor antagonist CNQX abolished network bursting and reduced synchronization of neuronal firing. Conversely, the addition of the GABA antagonist PTX to GN cultures increased the number of bursts and synchronization of neuronal firing as expected, while treatment with NMDA increased the mean firing rate (MFR; Figure 1C).

We then longitudinally measured and analyzed the mwMEA data of cultured GNs without pharmacological treatments. Over 3 weeks in culture, the total number of active electrodes did not change significantly (Figure 1D) suggesting that neuronal activity and, thus, neuronal connections between iPSC neurons appeared to be equally distributed across the entire well. However, over time and in particular, during the first few days in culture, an increase in the weighted MFR was evident (Figure 1E) concomitantly to the increased neurite growth in the first week after plating, followed by a plateau period afterward (compare timing to that in Figure 1B). Therefore, the initial increase in growth of GN neurites and branch points appeared proportional to the increased neuronal connectivity and spontaneous firing as measured by electrical network activity.

Burst frequency sharply increased after approximately 2 weeks in culture (Figure 1F). This is consistent with the literature (Tukker et al., 2018) and suggests that synchronized electrical network activity emerges delayed after a sufficient amount of neuronal connections has been established across an *in vitro* neuronal network. The synchrony index is another metric to quantify neuronal synchronization. This metric is based on measuring spike firing events between electrode pairs and comparing them across all electrodes throughout the mwMEA well (Paiva et al., 2010). In the commercially-available human iPSC neurons used in this study, the synchrony index steadily increased over time (Figure 1G) suggesting that firing progressively increased during the first 2 weeks and that neural networks mature over time. Thus, after approximately DIV14, relatively mature networks are established that are characterized by synchronous neuronal network burst activity. Taken together, our assessments demonstrate that for at least 3 weeks in culture, commercially-available GNs exhibit spontaneous neuronal activity and bursting behavior that can be leveraged to model human neuronal network development and function using mwMEA.

### Genome-Wide Gene Expression Dynamics in Cultured Human iPSC Neurons

We then aimed to better understand gene expression in cultured GN longitudinally on a genome-wide scale to investigate how GNs transcriptionally mature over time and to validate that GNs express genes relevant to the phenotypic readouts we are interested in, such as neurite dynamics and neuronal firing mentioned above. We monitored and compared the GN transcriptome *via* RNA-Seq after DIV7, DIV10, DIV14, DIV18, and DIV21. We first investigated the distribution of the genome-wide count data for all of the genes in the human transcriptome, broken down into quartiles (Figure 2A). and refer to genes within quartiles as having high, medium, and low levels of expression, respectively. Genes with no or very low expression (mean counts <5) were excluded.

Gene expression signatures of GN were compared to published postmitotic iPSC-derived neuron datasets which suggested a classification of iPSC-derived human neurons into less vs. highly mature and active types based on gene expression and electrophysiological features (Bardy et al., 2016). We determined expression levels of the top four genes specifically expressed in the highly functional AP5 human neuron type—*CKMT1B*, *GDAP1L1*, *PCLO*, and *TRAPPC6B—*longitudinally in GNs. All four genes were present in the highest gene expression quartile of all transcribed GN genes (Figure 2B) throughout the assayed 3-weeks-long cell culture period revealing that GN are postmitotic and properly differentiated human neurons and transcriptionally similar to highly functional AP5 type neurons. This is consistent with our mwMEA recordings (Figures 1E–H) and highlights the utility of GNs for electrophysiology studies in mature human neuronal cultures.

Next, to better understand how cultured GNs transcriptionally mature over time-periods which complement our longitudinal neurite dynamics and mwMEA datasets, genome-wide gene expression dynamics were compared between three key time points (DIV7, DIV14, and DIV21). Around DIV7 a plateau in neurite outgrowth becomes apparent and after ~DIV14 highly synchronous network activity is established, we terminated most experiments for this study at DIV21. Over 1,000 genes were differentially expressed when comparing DIV14 to DIV7 RNA-Seq datasets (536 upregulated, 526 downregulated; Figure 2C). An unbiased GO analysis further showed that differentially expressed genes were significantly enriched in the biological processes (BP) neuron differentiation and neuron projection development (Figure 2D). For example, the expression of key neuron differentiation genes steadily decreased over time (Figure 2E), which is consistent with our neurite dynamics data. The combined results suggest a shift to a morphologically and structurally mature state at DIV14 compared to DIV7. Between DIV21 and DIV14, almost 3,000 genes were differentially expressed (1,678 upregulated, 1,143 downregulated; Figure 2F). In particular, the genes involved in mRNA catabolism and translation were significantly enriched in the GO analysis (Figure 2G), demonstrating that during this time interval the human iPSC neurons are becoming functionally active as evidenced by the sharply increasing expression level of genes representative for the “Translation” group after DIV14 (Figure 2H), which once again mirrored the increased neuronal activity and synchrony we identified using mwMEA (Figure 1G). In all, these transcriptional dynamics highlight relevant developmental stage changes that occur as the GNs progress through 3 weeks *in vitro*.

### Human iPSC Neurons Express Key Neurodegenerative Disease-Linked Genes

A potential utility of these cells could be to facilitate the study of aberrant cellular functions in cellular models of human disease. Our RNA-Seq datasets to revealed that expression levels of AD-associated genes *APOE*, *APP*, *PSEN1*, and *PSEN2* were expressed consistently in the medium-to-high quartiles (Figure 3A), with *APP* being the most highly expressed disease gene. Relevant Parkinson’s Disease (PD)-associated genes *PARK7*, *PINK1*, *SNCA*, and *PRKN* are expressed at medium-to-high levels, while *LRRK2* is expressed at overall lower levels (Figure 3A). Finally, amyotrophic lateral sclerosis (ALS)-linked gene, including *SOD1*, and genes causing FTD, including *GRN*, are also robustly expressed in GNs at all assayed time points (Figure 3A).

### Progranulin Mediates Neuroprotection in Human iPSC Neurons

Next, we used GNs to understand the quantitative pharmacology of genes with an established role in neurodegenerative diseases in humans. PGRN is a pleiotropic growth factor that modulates diverse BP including neuronal survival and neurite outgrowth, PGRN mutations cause FTD (Van Damme et al., 2008; Gao et al., 2010; Gass et al., 2012). We analyzed the expression of the putative PGRN receptors (Figure 3B; Hu et al., 2010; Tang et al., 2011; Neill et al., 2016) in GNs. While the expression of *EPHA2* was relatively low, both *SORT1* and *TNFRSF1A* were highly expressed in GNs through the assayed 3 weeks in culture (Figure 3B). To qualify them as potential reference molecules and controls for our phenotypic assays, we verified that the expression of growth factors that have been shown to mediate neuroprotection (Nguyen et al., 2009, 2010) and enhance neurite outgrowths (Drubin et al., 1985; Iwasaki et al., 1998), such as brain-derived neurotrophic factor (BDNF) and nerve growth factor (NGF), BDNF and NGF and their signaling receptors (NTRK1, NTRK2, NTRK3, and NGFR), are robustly expressed in GNs (Figure 3C).

Cortical neurons from an FTD patient carrying mutated *GRN* show increased vulnerability to the neurotoxin staurosporine (Almeida et al., 2012), while the affected growth of iPSC-derived cortical neurons in a separate PGRN-FTD model is rescued by the reinstatement of *GRN* expression (Raitano et al., 2015). *in vitro* rodent studies have established that recombinant PGRN may promote neuronal survival (Van Damme et al., 2008; Ryan et al., 2009; Gao et al., 2010; Guo et al., 2010; Kleinberger et al., 2010; Xu et al., 2011). To complement rodent-derived data, we next quantified the responsiveness of human iPSC-derived GNs to recombinant PGRN in a neurotoxicity assay. GNs were grown uninterrupted for 2 weeks before being treated with 0.1 μM staurosporine (STS), an established non-selective protein kinase inhibitor and potent neurotoxin (Rüegg and Burgess, 1989), to trigger apoptotic neuronal death (Koh et al., 1995). To assess neuroprotection, cells were co-treated with either vehicle (PBS), positive controls (recombinant NGF or BDNF; Ichim et al., 2012), or a range of recombinant PGRN concentrations (Figure 4A) while cells were continuously monitored the fluorescence of the added annexin V dye to quantify apoptosis in our live-cell imaging platform. There were considerably more annexin V-labeled cells and annexin V-labeled area after 48-h STS treatment compared to treatment with medium alone, thus confirming STS cytotoxicity and validating the annexin V reagent to track cell death longitudinally (Figure 4B). Co-treatment of cells with either BDNF or NGF significantly lowered STS-induced neuronal death. Similarly, co-treatment experiments with PGRN concentrations of 0.114 nM or higher lead to a significant reduction in cell death compared to treatment with STS alone (Figures 4B,C). Our data complement evidence derived from rodent neurons and demonstrate a neuroprotective role for PGRN also in human GNs.

### No Sensitivity to Growth Factor-Mediated Neurite Dynamics in Human iPSC Neurons

We established a live-cell imaging neurite outgrowth assay using BDNF and NGF as positive controls (Figure 4D; Huang and Reichardt, 2001) to test if recombinant PGRN promotes neurite outgrowth in human GNs, as previously found in rodent neurons (Van Damme et al., 2008; Gao et al., 2010; Wang et al., 2010; Gass et al., 2012; De Muynck et al., 2013). Cells were incubated with recombinant growth factors or PGRN for 10 days, then morphological neurite metrics were analyzed (Figure 4D). BDNF, NGF (Iwasaki et al., 1998; Colombo et al., 2014), and PGRN (Van Damme et al., 2008; Gao et al., 2010; Wang et al., 2010; De Muynck et al., 2013) treatments using concentrations that have been previously shown to increase neurite outgrowth did not affect GNs (Figures 4C,G,H). Only 1,140 nM PGRN, which is ~16.000 times higher than the normal PGRN levels in human CSF (Nguyen et al., 2013), significantly enhanced neurite length in GN cultures. Cell-body cluster number was overall unchanged (Figure 4I), suggesting that captured effect sizes were not masked by variations in plating density, and thus neurite outgrowth in GNs may not be sensitive to physiologically-relevant concentrations of exogenous growth factor proteins.

### Acute PGRN Treatment Increases Neuronal Activity in Human iPSC Neurons

PGRN plays a role in synaptic plasticity (Tapia et al., 2011; Lui et al., 2016), PGRN treatment in murine cortical neurons can modulate aspects of spine maturation and synaptic transmission (Zhang et al., 2017). However, electrophysiological data in human iPSC neurons is lacking. To address this, we cultured GNs on mwMEA plates and exposed them to recombinant PGRN. mwMEA recordings were performed before, during, and immediately after treatment of functionally mature DIV19 GNs, followed by a final recording session 2 days after treatment (Figure 5A). Acute, one-time addition of 0.114 nM PGRN induced a significant increase in the overall firing activity of the cells, measured by the weighted MFR compared to the vehicle (Figures 5B,C). Surprisingly, 2 days after treatment, cells treated with either 0.00114, 0.114, or 11.4 nM PGRN also exhibited an enhanced firing rate compared to vehicle-treated cells (Figure 5C). Burst frequency was not modulated by the addition of PGRN either before or immediately after the washes, but 2 days after treatment a significant increase was observed for GNs that were exposed to 0.114 nM PGRN (Figure 5D). The network burst percentage, a measure of the spontaneous synchronization of burst activity across the network in the well, did not immediately respond to treatments but exhibited a significantly higher burst percentage after the washes up until 2 days after treatment (Figure 5E). The synchrony index (an alternative metric of neuronal synchronization) was not changed significantly, but 0.114 nM PGRN addition showed a trend towards increases network synchrony (Figure 5F). These results suggest that recombinant PGRN can enhance network activity in human iPSC neurons in a sustained manner.

### Using Human iPSC Neurons to Model Frontotemporal Lobe Dementia

To complement our approaches utilizing increased levels of (recombinant) PGRN, we next aimed to develop human cellular assays with decreased PGRN expression using siRNA (Baker et al., 2006; Cruts et al., 2006). First, we tested three different pools of self-delivering siRNAs in GNs, and found that in each tested pool we produced nearly complete knockdown of their target at 72 h (Figures 6A–D). To assess possible off-target effects of long-term siRNA exposure on neuronal health, morphology, and outgrowth, GNs were treated with a range of non-targeting (NT) siRNA concentrations, and their morphology was quantified. The highest concentration of NT siRNA tested (1,000 nM) caused extensive clustering of GNs at DIV21 (Figures 6E,H) and impeded neurite growth, but the tested lower concentrations of NT siRNA were well tolerated (Figures 6F–H).

We then studied the impact of various levels of GRN siRNA on *GRN* mRNA expression at DIV21 (Figure 7A). *GRN* mRNA was nearly undetectable in cells treated with 300 nM siRNA. A log-logistic dose-response model was fit to the *GRN* siRNA data (top = 1.073 ± 0.034, slope = 0.941 ± 0.111, ED_50_ = 10.45 ± 1.43 nM). This model indicated that 10 nM siRNA produced ~50% knockdown at DIV21, further experiments showed that 30 nM siRNA produced ~50% knockdown at DIV10 (Figure 7B). As expected in our approach to continuously replenish siRNA, the effects of 30 nM siRNA were more pronounced at the endpoint (71 ± 5.8% knockdown). We chose to continue using 30 nM siRNA for our experiments because it produced an approximated GRN haploinsufficiency in the time window that was most-relevant for our phenotypic assays (between ~DIV10 and ~DIV15) without noticeable off-target effects (Figures 6F–H).

### *GRN* Knockdown Does Not Impact the Transcriptome in GNs

Global gene expression abnormalities are present in human post mortem GRN-FTD brain samples (Chen-Plotkin et al., 2008). Next, we used RNA-Seq to determine if additional genes were changed in the GNs treated with 30 nM *GRN* siRNA or NT siRNA for up to DIV21 (Figure 7B). A PCA was used to reveal factors that modulate gene expression variance among samples. The first identified principal component was DIV, which accounted for 26% of the variance in the data (Figure 7C). This is not surprising given the expected developmental changes in gene expression (Figures 2C,F). The second principal component was the lot of GNs, which accounted for 8% of the variance (Figure 7D). The very little variance was due to the presence of either siRNA itself, as data from NT siRNA-treated cells were grouped with that from untreated cells. Surprisingly, knockdown of *GRN* also had little impact in the PCA (Figure 7C), which suggests that reduced *GRN* expression did not significantly affect the GN transcriptome. This was confirmed by pairwise analyses of the NT siRNA and *GRN* siRNA sample groups at DIV7, DIV14, and DIV21 (Figures 7E–G). This unbiased analysis detected significant decreases in GRN mRNA at DIV 14 and 21, which validated the RT-PCR data shown in Figure 7B and demonstrated that the study was powered to detect similar changes in other genes if they existed. We conclude that siRNA-mediated *GRN* knockdown in GNs under the current culture conditions did not affect global gene transcription signatures.

### *GRN* Knockdown Does Not Affect Neurite Dynamics in Human Neurons

**Figure 8:**
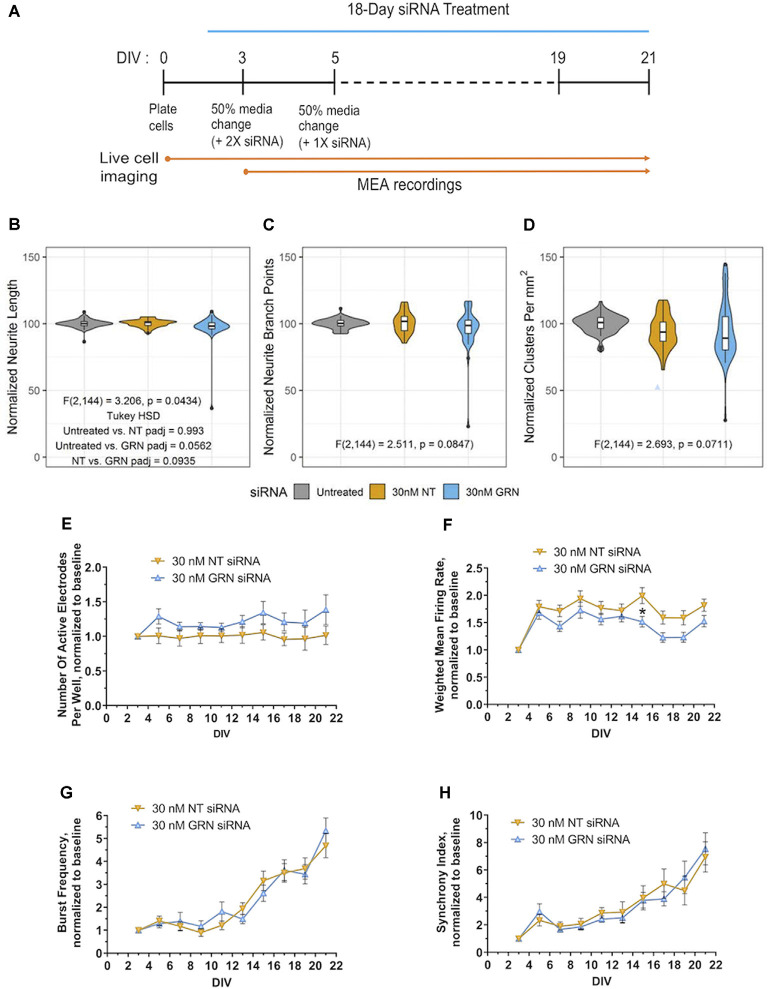
Morphological and electrophysiological features in human iPSC neurons with reduced *GRN* expression. **(A)** Schematic shows the repeated siRNA treatment protocol for knocking down GRN mRNA in GNs and the time course of the 21 DIVs with life cell imaging and mwMEA recordings. **(B–D)** Violin plots of GN neurite length **(B)**, branch points **(C)**, and cell-body clusters **(D)** at DIV21 following repeated treatment with the indicated concentrations of siRNA for 18 days. The white box plots display the median, with whiskers displayed using the Tukey method. Outliers are shown as black dots. The colored violin plots display the distribution of the data collected from 11 independent experiments (*n* = 69 untreated wells, *n* = 36 NT siRNA wells, and *n* = 42 GRN siRNA wells). The results of ANOVA are displayed in the graph along with the results of Tukey’s HSD test when the ANOVA results were significant. **(E–H)** Quantification of neuronal activity in GNs treated with either NT or *GRN* siRNA 30 nM from DIV3 to DIV21. **(E)** Graph showing active electrodes per well over 21 days in culture. **(F)** Plotted weighted MFR over 21 days in culture, normalized to weighted MFR measured at 3 DIV. **(G)** Graphed burst frequency over 21 days in culture, normalized to burst frequency recorded at DIV3. **(H)** Synchrony index over 21 days in culture, normalized to synchrony index recorded at 3 DIV. mwMEA graphs represent data from seven independent experiments (8–24 replicates per condition in each experiment). mwMEA graphs represent data from six independent experiments (two to six replicates per condition in each experiment), results are displayed as mean ± standard deviation (SD) *p < 0.05; (one-way ANOVA with Dunnett’s *post hoc* test, all conditions compared to NT siRNA).

Full *GRN* knockout rodent cortical and spinal cord neurons have decreased outgrowth and neurite complexity *in vitro* (Van Damme et al., 2008; Gao et al., 2010; Wang et al., 2010; De Muynck et al., 2013). Some neurons, including in the amygdala, may also show reduced neurite complexity in 6–9-month-old heterozygous *GRN* knockout mice *in vivo* (Arrant et al., 2016). We investigated next if the neurite dynamics are affected in cultured iPSC-derived human GNs with reduced PGRN expression. Automated and unbiased quantitative assessment of morphological features (illustrated by the schematic in Figure 8A) revealed that treatment of GNs with 30 nM of *GRN* siRNA had no significant effect on neurite length, neurite branch points, and density or movement of cell body clusters, compared to NT siRNA or untreated control cells (Figures 8B–D). *GRN* knockdown results mirror those from the recombinant PGRN treatment experiments described earlier (Figures 4G–I) and suggest that neurite dynamics in human iPSC-derived GNs under the probed conditions are not sensitive to bi-directional manipulations of progranulin levels.

### *GRN* Knockdown Does Not Consistently Affect Neuronal Network Activity in Human Neurons

By utilizing mwMEA recordings, we now explored if siRNA-mediated *GRN* haploinsufficiency in human GNs neurons modulates electrophysiological properties. The mwMEA activity of NT siRNA- and *GRN* siRNA-treated cells was longitudinally measured every second day starting at DIV3 (the first day of siRNA treatment) up to DIV21 (Figure 8A). Throughout the experiments, the number of active electrodes remained relatively constant. Although GRN siRNA-treated measurements trended lower, there was no significant difference between *GRN* siRNA- and NT siRNA-treated cells (Figure 8E). The weighted MFR in *GRN* siRNA-treated human iPSC neurons showed a consistent trend toward lower neuronal activity over 21 days that only reached statistical significance at DIV15 (Figure 8F). Consistent to previous experiments (Figure 1), the network burst frequency and synchrony index as a measure of neuronal synchrony increased over time in both NT siRNA- and *GRN* siRNA-treated cells, but no group difference was evident (Figures 8G,H). Our assessments of human iPSC neurons with reduced PGRN levels demonstrated no effect on global gene transcription or neuronal morphology and only modest modulation of neuronal firing. In all, our study describes a longitudinal systems-level characterization of GNs and highlights the diversity of phenotypic screening assays and manipulations of target genes that can be performed using these particular commercially-available human iPSC neurons.

## Discussion

Animal-based cellular models drive our understanding of disease biology, but often these tools do not have sufficient translational value for more clinically-relevant human biology. Therefore, in this study, we focused our attention on iPSC-derived neuronal cells as a key technology that is commercially available and, thus, would allow independent validation and wide distribution of the presented tools among the science community to open up this technology also to researchers that are not proficient in iPSC reprogramming. Since comprehensive and longitudinal profiling of human iPSC GNs is lacking, we have characterized systems-level phenotypic neuronal features (neurite outgrowth dynamics, neuronal network function, genome-wide gene expression).

Our longitudinal morphological dataset as a proof-of-principle demonstrates that live-cell imaging of GNs qualifies assay conditions and time points that are appropriate for investigating a given biological question. Furthermore, quantitative pharmacology of compounds that potentially modulate a cellular process can be performed using the assays and conditions outlined in this study. For example, since neurite outgrowth reaches a plateau around ~DIV10, GN experiments that focus on studying these neurite dynamics during the phase of strongest growth should be performed before DIV10.

To complement the quantification of morphological features in GNs, we established a long term mwMEA platform that enabled us to quantify the formation and synchronization of functional neuronal network activity in human iPSC neurons. Our results are consistent with previous studies in both rodent and human neurons (Tukker et al., 2018; Odawara et al., 2016; Tukker et al., 2016) and demonstrate that, as neurons grow *in vitro*, they spontaneously form networks that are characterized by electrical activity. Our data further revealed that GNs need approximately 2 weeks in culture before a robust synchronization of neuronal firing develops across the well (Figure 1). Therefore, human iPSC GNs are a valuable tool to study a variety of electrophysiological features and offer ways to robustly quantify (either acutely or over time) how neuronal firing and synchrony may be modulated by genetic or pharmacological manipulations.

The third major arm of longitudinal phenotypic characterization that we performed in GNs was a comprehensive genome-wide gene expression characterization spanning 3 weeks in culture. Our data validate that GNs are of a postmitotic neuronal phenotype and that intrinsic global gene expression is fluid during the *in vitro* culture period. While the first week of culture (growth phase) is characterized by genes that are typically expressed during neuronal development, the second week (maturation and refinement phase) is dominated by neuronal differentiation gene networks. The third week of culture (synchronous and steady-state phase) shows highly expressed gene networks typical for very mature neuronal homeostasis and activity (Figure 2). These changes in the gene expression profile over time are consistent and overlap with the developmental progression of the previously quantified morphological and functional features in GNs (Figure 1). Besides, our RNA-Seq data also can be utilized for data mining in the context of identifying molecular targets and genes that are associated with neuronal diseases. We show, for example, that GNs express relevant genes that are important for studying growth factors or neurodegenerative diseases including Alzheimer’s, Parkinson’s, ALS, and FTD (Figure 3). To allow additional analyses of our data, data sharing, and combination with other published datasets, our RNA-Seq datasets will be made publicly available at the Gene Expression Omnibus as a resource to study and better understand the longitudinal expression of any gene(s) of interest in GN cultures.

To provide a practical demonstration, we have leveraged our GN datasets to investigate relevant human neurobiology questions in phenotypic assays. For example, we have investigated if growth factor treatment during the initial neuronal outgrowth phase impacts the dynamics of neurite outgrowth; in separate assays, we have quantified if such treatment can mediate neuroprotection and lower apoptotic cell death. One of the key findings of our study is that recombinant progranulin is neuroprotective in human neurons (Figure 4). This is noteworthy for at least two reasons: (1) revealing that the potency of PGRN to protect human neurons against apoptosis caused by STS (Almeida et al., 2012) is comparable to that of BDNF and NGF (Aloe et al., 2012; Allen et al., 2013; Kowiański et al., 2018); and (2) this result builds a translational bridge from previous rodent neuronal data (Van Damme et al., 2008; Gao et al., 2010; Gass et al., 2012; Wang et al., 2010; De Muynck et al., 2013) to human neuronal data.

Some involvement of PGRN in synapse biology is prominent in the literature (Tapia et al., 2011; Petoukhov et al., 2013; Yuan et al., 2015; Lui et al., 2016; Meeter et al., 2016; Zhang et al., 2017; Uesaka et al., 2018), but not much was known about the role of PGRN in regulating neuronal firing. Our mwMEA experiments provide evidence that a one-time acute treatment with recombinant PGRN can increase neuronal firing and synchrony in human iPSC neurons (Figure 5). Surprisingly, we further found that this effect appears to be long-lasting for at least up to 2 days after treatment. While we currently do not understand which mechanism mediates this activity, future studies that investigate the role of PGRN are warranted including for example to complement our existing data with PGRN overexpression or GRN gene editing using e, g, CRISPR technologies.

Our proof of principle approach to model FTD in human neurons *in vitro* using siRNA-mediated *GRN* knockdown demonstrated that GNs combined with other off-the-shelf reagents can be used to elucidate the functions of disease-associated genes in highly customized isogenic models and assays of human disease (Figure 6). At this stage, our analyses of this model are still preliminary and suggest that siRNA-mediated *GRN* knockdown in GNs under the current culture conditions seemingly did not modulate global gene transcription signatures (Figure 7), although gene expression defects have been reported in GRN-deficient post mortem brains (Chen-Plotkin et al., 2008). We speculate that a *GRN* knockdown of longer than 2–3 weeks could be needed to influence the genome-wide transcriptional landscape more robustly in cultured GNs. It is further difficult to directly compare our human GN knockdown model, which was designed to mimic haploinsufficiency and have a ~50% reduction in PGRN levels to previous studies utilizing PGRN-deficient mice. Virtually all phenotypes described previously in rodent neurons were derived from analyzing full PGRN knockout tissues or cells, which do not express PGRN at all. Additional experiments are needed to more deeply investigate PGRN biology in human cellular models, our study provides a validation of available siRNA tools and a roadmap to demonstrate how our isogenic and scalable knockdown protocol can be leveraged to manipulate the expression of virtually any gene in the genome in commercially-available human iPSC GNs. In conclusion, we have deeply characterized one commercially-available human iPSC-derived neuronal cell line as a potential translatable tool for fundamental and translational neuroscience research.

## Data Availability Statement

RNA-Seq data are publicly accessible at the Gene Expression Omnibus under Accession GSE157573.

## Author Contributions

GR, JE, and DH performed research, analyzed data, and wrote the article. PW designed research and wrote the article. AZ designed research, analyzed data, and wrote the article. All authors contributed to the article and approved the submitted version.

## Conflict of Interest

PW was employed by the company GlaxoSmithKline (GSK) and AZ was employed by the company Memento Therapeutics Corporation. The remaining authors declare that the research was conducted in the absence of any commercial or financial relationships that could be construed as a potential conflict of interest.
